# A Nano-Rattle SnO_2_@carbon Composite Anode Material for High-Energy Li-ion Batteries by Melt Diffusion Impregnation

**DOI:** 10.3390/nano10040804

**Published:** 2020-04-22

**Authors:** Sivarajakumar Maharajan, Nam Hee Kwon, Pierre Brodard, Katharina M. Fromm

**Affiliations:** 1 Department of Chemistry, University of Fribourg, Chemin du Musée 9, CH-1700 Fribourg, Switzerland; sivarajakumar.maharajan@unifr.ch; 2 College of Engineering and Architecture of Fribourg, University of Applied Sciences of Western Switzerland, Boulevard de Pérolles 80, CH-1705 Fribourg, Switzerland

**Keywords:** SnO_2_@C, nano-rattles, anode, Li-ion batteries

## Abstract

The huge volume expansion in Sn-based alloy anode materials (up to 360%) leads to a dramatic mechanical stress and breaking of particles, resulting in the loss of conductivity and thereby capacity fading. To overcome this issue, SnO_2_@C nano-rattle composites based on <10 nm SnO_2_ nanoparticles in and on porous amorphous carbon spheres were synthesized using a silica template and tin melting diffusion method. Such SnO_2_@C nano-rattle composite electrodes provided two electrochemical processes: a partially reversible process of the SnO_2_ reduction to metallic Sn at 0.8 V vs. Li^+^/Li and a reversible process of alloying/dealloying of Li*_x_*Sn*_y_* at 0.5 V vs. Li^+^/Li. Good performance could be achieved by controlling the particle sizes of SnO_2_ and carbon, the pore size of carbon, and the distribution of SnO_2_ nanoparticles on the carbon shells. Finally, the areal capacity of SnO_2_@C prepared by the melt diffusion process was increased due to the higher loading of SnO_2_ nanoparticles into the hollow carbon spheres, as compared with Sn impregnation by a reducing agent.

## 1. Introduction

The current commercial anode materials for Li-ion batteries, graphite and Li_4_Ti_5_O_12_, have a low capacity (375 and 175 mAh g^−1^, respectively). Furthermore, graphite has safety problems due to lithium plating, causing a short circuit and damage of batteries. [1,2] Alloy anodes such as Li_x_Sn or Li_x_Si possess theoretical specific capacities of 994 and 3579 mAh g^−1^ for Li_4.4_Sn and Li_15_Si_4_, respectively, which are at least three times higher than that of graphite and Li_4_Ti_5_O_12_. [3,4,5,6] The potential of alloy anode materials is 0.3–1.0 V vs. Li/Li^+^ higher than that of graphite (0.05 V vs. Li/Li^+^) [4,5] but lower than that of Li_4_Ti_5_O_12_ anodes (1.5 V vs. Li/Li^+^) [3]. Such potentials are very advantageous with respect to the safety of Li^+^ ion batteries. The main hurdle for the commercialization of these alloy anodes is, however, the huge (non-uniform) volume variation of up to 300% during Li^+^ insertion and extraction, leading to the rupture of the active alloy particles, and hence, poor electrical contact, poor cycling and capacity fading [7].

To overcome the rapid capacity drop of Li*_x_*Sn alloys, core-shell Sn@carbon was proposed. One of the synthetic approaches to make a core-shell nanostructure is that tin ions are reduced in the solution of carbon precursors by using reductive agents such as reductive gases, alkali metal borohydrides or hydrazine. [8,9,10,11,12]. However, reduced metallic tin can be lost in core-shell Sn@C composite during high temperature carbonization due to its low melting point of 232 °C [11].

The SnO_2_-based anode material places itself in a remarkable top position due to its high theoretical capacity of 1491 mAh g^−1^. It is also non-toxic and not very expensive in terms of commercialization. [7] As a drawback, SnO_2_-based anode materials experience a drastic volume change of 360% due to the expansion and contraction accompanying lithiation and delithiation, respectively [13]. This results in severe mechanical disintegration of the anode particles and in pulverization of the active material and subsequent capacity loss [14,15].

To overcome the pulverization of SnO_2_ during the reaction with Li^+^ ions, hollow SnO_2_ can be obtained via template-free synthesis with a good cycle life due to the presence of the hollow nanostructures [16,17]. As another example, nanostructured SnO_2_ electrodes [18,19,20,21,22,23,24,25] and 200 nm hollow SnO_2_ nanoparticles, relying on the large surface area, improved the structural stability and short Li^+^ ion diffusion path lengths. [26] Despite these advantages of nanostructured SnO_2_, there are short-comings, including the low packing density and an unsatisfying cycle life [26].

Most recently, carbon has been used in a nanocomposite approach to prepare ‘active element/inactive matrix’ setups, where carbon is considered as an inactive matrix whose electrochemical performance is negligible [10,27]. Core-shell SnO_2_@C nanostructures were obtained via encapsulation of SnO_2_ active material in hollow carbon spheres using the sacrificial templating method. [28] This strengthened the structural integrity of the active material and improved the capacity due to the enhanced SnO_2_ loading into the hollow carbon spheres [28,29,30]. In addition, the carbon-based inactive matrix served as a buffer for the volume change, reducing the consequential pulverization [28,31,32].

However, the synthesis of monodispersed hollow carbon through soft-templating methods and a high loading of SnO_2_ nanoparticles on the hollow carbon are still great challenges that remain [29]. Furthermore, the morphology and size of the SnO_2_ particles affect the volume changes during lithiation [33,34,35,36]. Hence, the size and the distribution of SnO_2_ particles in the carbon matrix are critical to reach a high performance. Herein, we report the synthesis of SnO_2_@C nano-rattles achieved by melt diffusion and the battery properties observed using this material as an anode material.

## 2. Materials and Methods

**Figure 1:**
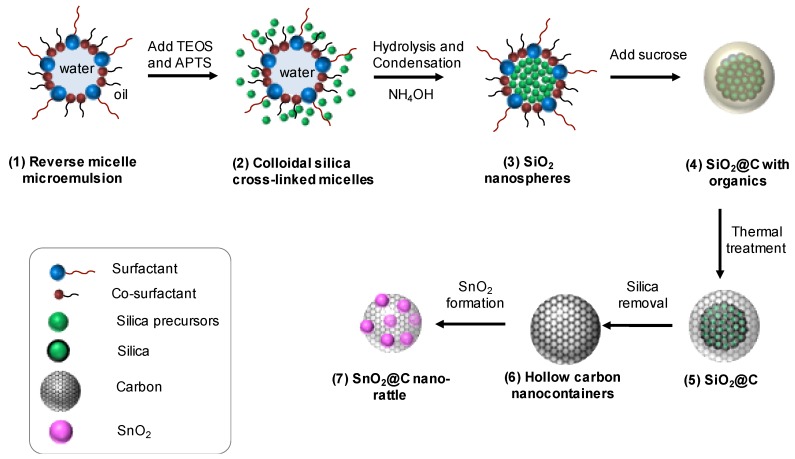
Schematic illustration of the formation of SnO_2_@C nano-rattles. (1) Reverse micelle microemulsion, (2) Colloidal silica cross-linked micelles, (3) SiO_2_ nanospheres, (4) SiO_2_@C with organics, (5) SiO_2_@C, (6) Hollow carbon nanocontainers, (7) SnO_2_@C nano-rattles.

Figure 1 resumes the synthetic process used to obtain the SnO_2_@C nano-rattles. Each step was optimized after testing different conditions.

### 2.1. Synthesis of the Nanospherical Silica Template

The procedure has been adapted from the reverse micelle microemulsion technique [37]. The reverse micelles are formed by the addition of 10 g of Triton X-100 (surfactant) to 29.6 g of cyclohexane, followed by the addition of 8 mL of hexanol (co-surfactant). After 2 h of vigorous and homogeneous mixing, 1.7 mL of ultrapure MilliQ water is added to the as-formed reverse micelle system. After another 2 h, 200 μL of TEOS (tetraethyl orthosilicate) is added to the solution followed by the addition of 50 μL of 12.5 v/v% ethanolic solution of APTS (3-aminopropyl triethoxysilane). Adding 25% NH_4_OH led to the hydrolysis and polycondensation of silica precursors in reverse micelles during 2 h. After thoroughly dispersing and washing the obtained product with ethanol three times and twice with water, the desired SiO_2_ nanospheres have been obtained. The yield was found to be ~ 40%.

### 2.2. Formation of Hollow Carbon Spheres

To obtain the hollow carbon spheres, sucrose was used as the carbon precursor with a weight ratio of 1:4 for SiO_2_:sucrose. A hydrothermal carbonization in aqueous medium was carried out at 180 °C for 20 h.

To remove residual impurities from the hydrothermal carbonization, the as-obtained carbon coating was thermally treated under argon in two steps, first heating to 400 °C at a rate of 1 °C/min with dwelling for 3 h, then heating to 750 °C at 5 °C/min, keeping this temperature for 3 h. To remove the silica template, the material was soaked in 10% aqueous HF for 48 h, yielding the desired hollow carbon spheres. The yield was found to be ~ 68%.

### 2.3. SnO_2_@C Nano-Rattles via Melt Diffusion

The desired SnO_2_@C nano-rattles were obtained by impregnating Sn nanoparticles into the carbon shell using the melt diffusion method. The Sn precursor (SnCl_2_·2H_2_O, melting point 37.7 °C) was hand-mixed in a mortar with the hollow carbon spheres for 30 min and then fed into a tube furnace for the melt diffusion process. The mixture was heated in air to 150 °C at 2 °C/min for 2 h, heated to 250 °C at a rate of 5 °C/min, kept there for 2 h overall, and finally dwelling for 6 h at 350 °C using a heating rate of 5 °C/min in order to form the SnO_2_@C nano-rattle composite.

### 2.4. Sn@C Nano-Rattles via Wet Impregnation

To compare the performance with SnO_2_@C electrode, Sn@C was synthesized under Ar atmosphere using a Schlenk line; 0.1 M of dried anhydrous SnCl_2_ was dissolved in 50 mg of the as-obtained carbon dispersed 8 mL of diethylene glycol. After sonication of the solution for 5 min to disperse the Sn precursors in the solution, the solution was agitated overnight for 12 h to homogeneously adsorb into the hollow carbon spheres. Two milliliters of 0.1M NaBH_4_ were injected into the solution drop by drop to reduce the Sn^2+^ ions to metallic Sn nanoparticles in the carbon shell. The molar ratio of SnCl_2_:NaBH_4_ was 1:1.11.

### 2.5. Characterization

Scanning Electron Microscopy (SEM, Philips XL30, 10–15 kV, Philips, Eindhoven, Netherlands) and Transmission Electron Microscopy (TEM, FEI/Philips CM-100, 80 kV, Philips/FEI Corp., Eindhoven, Netherlands) were performed to study the morphology and size of the particles. The TEM samples were prepared by dropping a diluted and sonicated suspension onto the copper TEM grids (Electron Microscopy Sciences, CF 300-Cu, Carbon Film on 300 Square Mesh Copper Grids). An X-ray Electron Dispersive Spectroscopy (EDS) analysis coupled with SEM was performed to elucidate the elemental composition of the material of interest. The phases of the materials were identified by X-ray diffraction (XRD, Cu-radiation, STOE, Darmstadt, Germany) analysis. The XRD data were collected from 2 theta = 10 to 80° at a scan rate of 0.05° per step and 30 s per point. Fourier Transform-Infrared (FT-IR) spectroscopy was carried out to analyze the functional groups in the material. The IR spectra were collected in the range of 400 to 4000 cm^−1^ using attenuated total reflectance method. Thermogravimetric analyzer (TGA) (Mettler Toledor, Giessen, Germany) was employed to correlate the weight loss associated with thermal changes under air until 1000 °C at 10 °C/min. Brunauer–Emmett–Teller (BET, ASAP 2010, Micromeritics, Unterschleissheim, Germany) nitrogen absorption method was used to measure the specific surface area and porosity information. Raman spectroscopy was applied with a confocal micro-Raman spectrometer (LabRAM HR800, HORIBA, Palaiseau, France) combined with an optical microscope Olympus BX41) using a red laser at 633 nm for excitation, attenuated with filters in order to avoid thermal degradation of the samples.

### 2.6. Preparation of the Electrodes

The SnO_2_@C nano-rattles were mixed with polyvinylidene fluoride (PVDF) as a binder and carbon (Super C65) as conductive additive in N-methyl-2-pyrrolidone (NMP) solvent. The weight ratio of SnO_2_@C nano-rattles, carbon additive and the binder was 77:15:8. The slurry was prepared in a sequence as the binder was mixed in the solvent for 30 min and then the carbon conductive additive and the SnO_2_@C nano-rattles were mixed sequentially for 30 min for each addition. The as-prepared slurry was deposited on a Cu current collector using an MSK-AFA-II Automatic Thick Film Coater (MTI, Richmond, USA). The electrodes were dried at 120 °C under vacuum over night for 12 h. The mass loading of SnO_2_ in the electrode was calculated to be 2 mg based on the analysis of TGA (refer supplementary information and Table S1). The specific capacity was calculated based on the mass of SnO_2_ in SnO_2_@C (refer supplementary information). The same procedure was followed to prepare the electrodes for Sn@C. The mass loading of Sn in Sn@C was calculated to be 1.68 mg.

### 2.7. Electrochemical Characterizations of Electrodes

The electrodes were assembled into a coin cell with lithium metal as the counter electrode, a 1:1 volume ratio of a mixture of ethylene carbonate (EC) and dimethyl carbonate (DMC) with 1 M LiPF_6_ as the electrolyte, and a Celgard membrane as separator. The coin cell assembly was performed in an Ar filled glove box (MBraun, Garching, Germany). Cyclic voltammetry (CV) technique was performed using a Princeton potentiostat within the potential range of 0.02–2.2 V vs. Li^+^/Li at scan rates of 0.05, 0.1 and 0.5 mV s^−1^. The charge/discharge characteristics were evaluated with an Arbin 2000 battery test instrument at the same potential range as CV. The charge/discharge current rate was calculated with respect to the capacity of SnO_2_ (781 mAh g^−1^) at various current densities (C/10, C/7.5, C/5, C/2 and C). The Coulombic efficiency was calculated as C_delithiation_/C_lithiation_, where C_delithiation_ and C_lithiation_ are the capacities during Li extraction and insertion, respectively.

## 3. Results and Discussion

Using reverse micelles (water-in-oil) is more efficient than micelles (oil-in-water) because the undesirable formation and random cross-linking leading to mesocellular foam can be avoided in the oil-continuous phase [38]. We used the surfactant Triton X-100, which has longer hydrophilic groups than Igepal CA-520, in order to produce a suitable diameter of the microemulsion reverse micelles (>50 nm. Figure S1). The co-surfactant *n*-hexanol helps to form a stable microemulsion [39]. In addition, using Triton X-100 and *n*-hexanol can make more monodispersive microemulsions than using solely Igepal CO-520 [40,41]. As silica precursors, two precursors TEOS and APTS were used in order to obtain hollow silica spheres [40]. The reverse micelle microemulsion was firstly prepared upon the addition of water into a mixture of cyclohexane and surfactants (Figure 1(1)). Subsequently, the two silica precursors TEOS and APTS were added to form colloidal silica micelles (Figure 1(2)). The formation of silica nanoparticles was initiated by the hydrolysis and the poly-condensation process after the addition of ammonia solution (Figure 1(3)). Sucrose was then coated on the surface of the silica particles and decomposed using hydrothermal carbonization (Figure 1(4)). A subsequent thermal treatment was carried out to form the final carbon coating (Figure 1(5)). The silica was finally removed to create hollow carbon spheres (Figure 1(6)), into which SnO_2_ was impregnated by melt diffusion using tin chloride dihydrate (Figure 1(7)). Figure S2 shows the color of the materials, respectively suspensions at each step: white for colloidal silica suspension, brown for the carbon coated silica after the hydrothermal process, black for carbon coated silica after thermal treatment, and black for carbon powder after the removal of silica.

**Figure 2:**
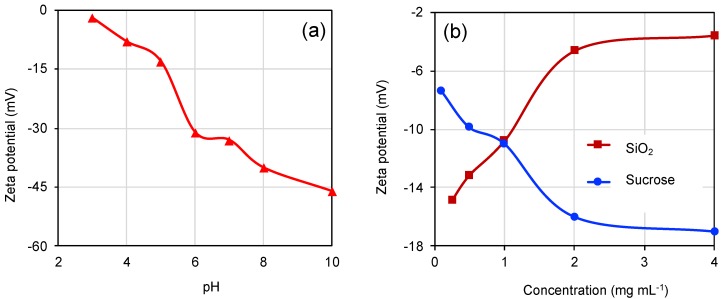
Graphs showing the zeta potential analysis of SiO_2_ with respect to pH (**a**) and concentration at pH 6 (**b**) to optimize the hydrothermal carbonization process.

In order to achieve a homogenous carbon coating of the silica nanoparticles, it was important to understand and control the colloidal silica with the carbon precursor [42,43]. The chosen carbon precursor sucrose is well known for its high carbon content and abundance [42]. A zeta potential measurement was performed to characterize the surface charge of the silica particles at different pH and concentration. It was shown to become increasingly negative upon increasing pH (Figure 2a). With increasing concentration of silica in the suspension, the zeta potential decreases, leading to an agglomeration of the silica in the suspension (Figure 2b). The optimal pH value was found to be around 6, leading to a negative zeta potential in the range of −30 mV and hence a stable suspension avoiding aggregation. Furthermore, a low concentration of silica together with an increasing sucrose concentration yielded a stable net negative zeta potential overall (sucrose and silica mixed solution), leading in our hands to an efficient carbon coating by hydrothermal carbonization, after which the initially colorless silica turned brown (Figure S2).

**Figure 3:**
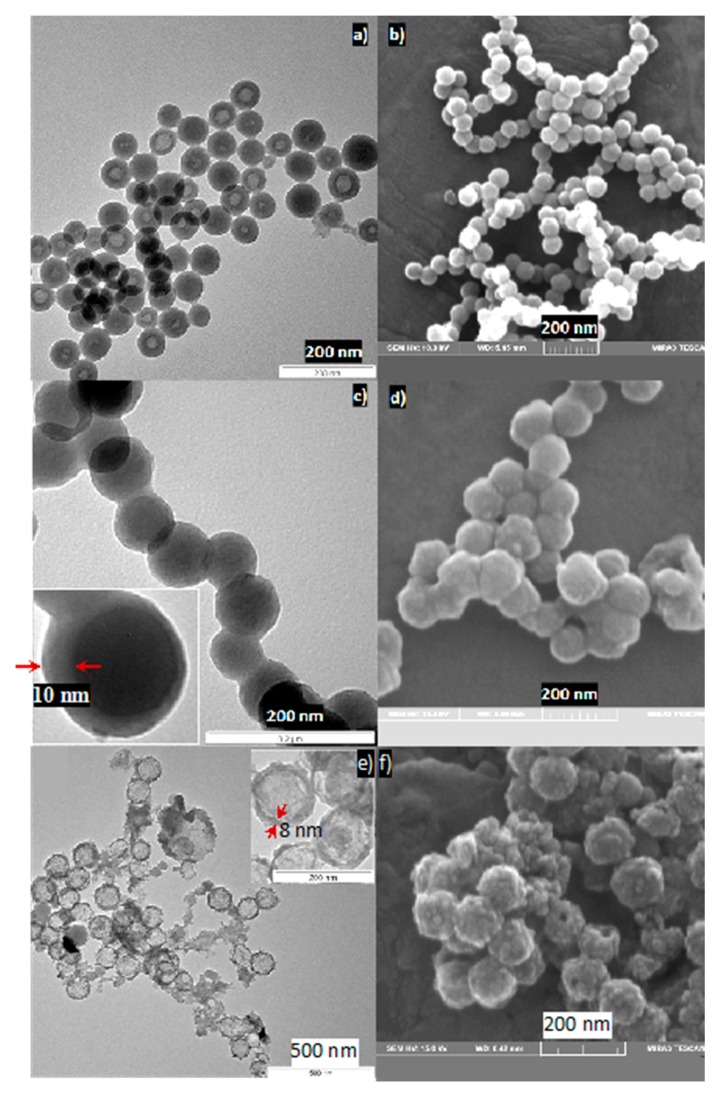
TEM (**a**,**c**,**e**) and SEM (**b**,**d**,**f**) images showing silica spheres after reverse micelle synthesis (**a**,**b**), SiO_2_@HTC after hydrothermal carbonization (**c**,**d**), and empty hollow carbon spheres after silica removal (**e**,**f**), respectively.

The morphologies of the bare silica template and the carbon-coated silica after hydrothermal carbonization (SiO_2_@HTC) have been investigated using SEM and TEM (Figure 3). The silica nanoparticles are in the size range of 60 to 80 nm. Apart from solid silica particles, the TEM image in Figure 3a shows also the formation of hollow silica spheres arising from partial or incomplete silica condensation at the core of the micelles [40]. This does however not influence the carbon coating. The TEM and SEM images (Figure 3c,d) confirm the formation of a uniform and homogeneous carbon coating on silica after the hydrothermal carbonization process, leading to particles of ca. 100 nm in diameter. Given the initial size of the bare silica, it can be assumed that the carbon coating is 10–20 nm thick.

After thermal treatment under argon atmosphere, the brown SiO_2_@C turned black (Figure S2). During the thermal treatment, residual oxygen-containing groups (hydroxyl, ester or ether) are destroyed and amorphous, sp^2^-hybridized carbon is formed, as shown by Raman spectroscopy (shown later). In order to form the hollow carbon spheres, the silica of the SiO_2_@C nano-spheres was etched in 10% aqueous HF solution for 48 h. The obtained carbon was porous with a wall thickness of ~8 nm (Figure 3e). The porosity of carbon is crucial to allow efficient etching of silica and SnO_2_ impregnation into hollow carbon spheres.

**Figure 4:**
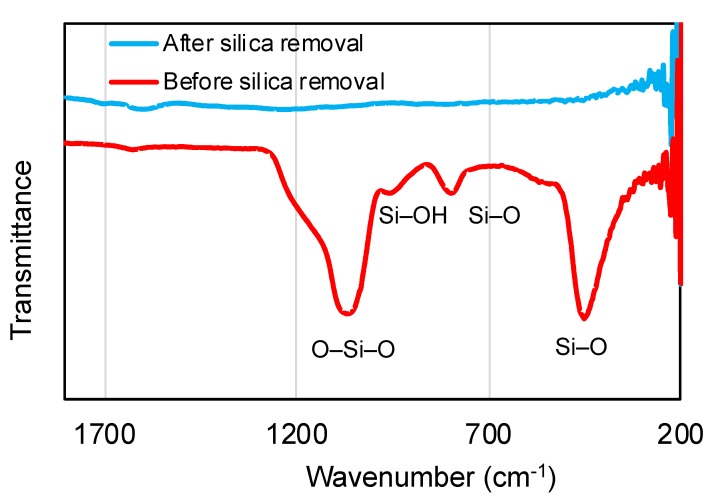
FTIR analysis showing the absence of silica after the etching process.

The Fourier transform infrared spectroscopic (FTIR) analysis further confirms the removal of the silica core (Figure 4) as the bands of silica disappeared completely after the etching.

The empty porous carbon spheres were used for a melt diffusion process [44] in order to form SnO_2_ nanoparticles within the carbon spheres via thermal decomposition of the SnCl_2_•2H_2_O precursor (Equation (1)). With a low melting point of 37.7 °C, the molten SnCl_2_·2H_2_O diffuses into the mesoporous carbon during the thermal heating steps [44]. The thermal decomposition and SnO_2_ formation happen in three steps; first water is removed at 180 °C, then SnO_2_ is formed at 280 °C and finally the generation of SnO_2_@C happens at 350 °C. The residual impurities were washed out carefully.

SnCl_2_∙2H_2_O + O_2_ → SnO_2_ + Cl_2_ ↑ + 2 H_2_O ↑ (1)

**Figure 5:**
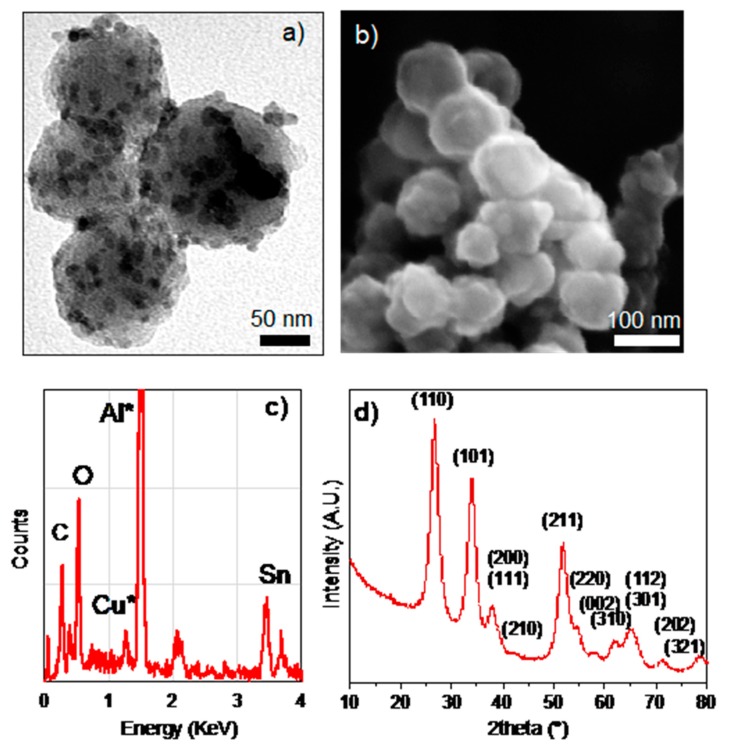
TEM (**a**) and SEM (**b**) images showing SnO_2_@C nano-rattle after melt diffusion; EDS spot analysis confirming the presence of Sn, O and C elements (**c**). XRD of after molten Sn precursor melt diffusion impregnation (**d**).

The as-formed SnO_2_@C nano-rattles are shown in Figure 5. Small dark dots with <10 nm of diameter inside the carbon spheres as well as a rough particle surface, different from the morphology of carbon shell alone without SnO_2_ (Figure 3e,f)), indicate the presence of tin. The EDS analysis confirmed the presence of tin from SnO_2_ in the carbon shells (Figure 5c), while XRD identified the presence of SnO_2_ (Figure 5d). Hence, the material was characterized as <10 nm sized SnO_2_ nanoparticles distributed uniformly inside and on the porous carbon spheres of about 80 nm.

**Figure 6:**
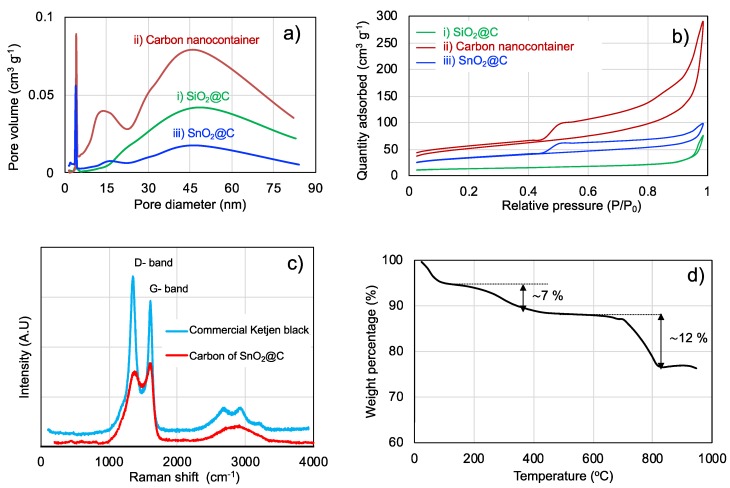
Pore distribution (**a**) and adsorption isotherms (**b**) of (i) SiO_2_@C before silica removal, (ii) hollow carbon nanocontainers after silica removal and (iii) SnO_2_@C nano-rattles after melt diffusion. Raman spectrum analysis of carbon structure (**c**) and TGA (**d**) of SnO_2_@C.

The porosity was studied using the N_2_ adsorption/desorption method for three samples, (i) SiO_2_@C and (ii) the carbon nanocontainer after silica template removal, and (iii) SnO_2_@C after the melt diffusion impregnation. Figure 6a,b and Table 1 show the pore size distribution, adsorption isotherm and specific surface area of these three samples. The adsorption hystereses in Figure 6b show that the shape of (i) is rather nonporous or microporous, while the samples of (ii) and (iii) are mesoporous with no limiting adsorption at high *P*/*P*_0_. SiO_2_@C had the lowest surface area (43 m^2^ g^−1^) among the three samples (i), (ii) and (iii). After the silica template removal from SiO_2_@C, the highest surface area was found for sample (ii), featuring three different pore sizes of 3 nm, 15 nm and 45 nm in diameter as shown in Figure 6a. The small pores of 3 and 15 nm are assumed to correspond to holes in the carbon shells. These pores are not present in the sample of (i) SiO_2_@C before the removal of silica. The 45 nm pores can be interpreted as the inner diameter of the carbon nanocontainers. After the formation of SnO_2_ nanoparticles in and on the porous carbon spheres, the pore size and the pore volume shrink, and the surface area of (iii) SnO_2_@C is reduced to 110 m^2^ g^−1^. Hence, the SnO_2_ evidently fills the hollow carbon nanocontainers as well as pores in the carbon, but likely not completely, yielding a rattle type assembly (Figure 6a).

The structure of carbon on SnO_2_@C nano-rattles has been characterized by Raman spectroscopy (Figure 6c). The obtained SnO_2_@C nano-rattles indeed show the typical disordered *D*-band and ordered *G*-band for C–C bonds at 1323 and 1620 cm^−1^, respectively. [25,45,46] These values correspond well with the data for commercial, highly porous amorphous Ketjen carbon black, confirming also the presence of *sp*^2^- hybridized carbon atoms. The *G*/*D* ratio was calculated as 0.76 by the peak areas of the *G* and *D*-bands, where a broader Raman peak is seen in the D-band. A value of <1 in the *G*/*D* ratio means that the disordered C–C bonds (*D*-band) attributed from out-of-plane vibrations are more dominant than the ordered ones (*G*-band) from in-plane vibrations. The *G*/*D* ratio of commercial Ketjen black was 0.83, which is also lower than 1.

The amount of carbon and SnO_2_ in the SnO_2_@C nano-rattles after hydrothermal treatment have been evaluated by TGA in air (Figure 6d). It was found that the material consists of ca. 76% w of SnO_2_, 12% of carbon and 7% of residual organic materials. The TGA analysis was followed by XRD analysis on the white residue, confirming the presence of crystalline SnO_2_.

**Figure 7:**
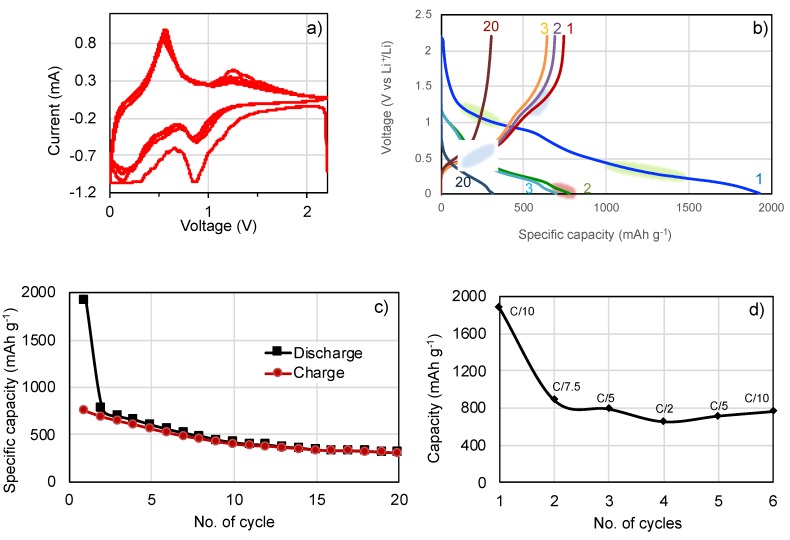
Cyclic voltammetry analysis of SnO_2_@C nano-rattles at 0.05 mV s^−1^ (**a**). Charge and discharge curves (**b**) and cyclability (**c**) of SnO_2_@C composite electrode at C/5 for 20 cycles. Rate capability test at different *C*-rates for coin cells with electrode based on SnO_2_@C nano-rattles (**d**).

The SnO_2_@C nano-rattles were used to fabricate electrodes for coin cells. The redox reaction of SnO_2_ was analyzed by measuring cyclic voltammograms in the range of 0.02 to 2.2 V vs. Li^+^/Li at a scan rate of 0.05 mVs^−1^ as shown in Figure 7a. The lithium storage mechanism of SnO_2_ occurs in two steps as follows [33,47,48].

SnO_2_ + 4 Li^+^ + 4 e^−^ ←→ Sn + 2 Li_2_O (2)

Sn + *x* Li^+^ + *x* e^−^ ←→ Li*_x_*Sn (0 ≤ *x* ≤ 4.4) (3)

In the first step (Equation (2)), SnO_2_ reacts with Li^+^ ions and electrons to metallic Sn and inactive Li_2_O, which is indicated by the cathodic peak at 0.8 V vs. Li^+^/Li in Figure 7a. The corresponding anodic peak at 1.3 V vs. Li^+^/Li corresponds to the oxidation of Sn to SnO_2_. The cathodic current at the first cycle was high and then reduced significantly at the following cycles, giving rise to a (partially) reversible activity. This conversion reaction of SnO_2_ to metallic tin is quasi reversible only for nanosized SnO_2_ while it is not reversible for bulk SnO_2_ [33,49,50].

In the second reaction (Equation (3)), metallic Sn alloys reverses with Li^+^, a process during which the volume of Li*_x_*Sn, 2 < *x* < 4.4 changes up to 200%. This reaction occurs at 0.2 V and 0.56 V Li^+^/Li for the cathodic and anodic peaks, respectively. This performance is in good agreement with the literature [31,32,51,52].

When these electrochemical processes are overall reversible, 8.4 Li^+^ are processed for one unit of SnO_2_, corresponding to 1491 mAh g^−1^, while if only the second step is considered reversible, 4.4 Li react per one unit of Sn, corresponding to 781 mAh g^−1^.

Overall, the redox reactions occurring in CV are (i) SnO_2_ ↔ Sn at higher potential (>0.8 V) and (ii) Sn ↔ Li*_x_*Sn at lower potential (~ 0.5 V).

Figure 7b shows the charge and discharge behavior of the SnO_2_@C electrode at C/5. The discharge capacity reached 1924 mAh g^−1^ at the first cycle (in blue no.1), which is higher than the theoretical value of 1491 mAh g^−1^ corresponding to insertion of 8.4 Li^+^ (Equations (2) and (3) above). The excessive capacity of 430 mAh g^−1^ based on 1491 mAh g^−1^ may result from the formation of the SEI layer on SnO_2_@C and Li^+^ storage in carbon during the first cycle [7,53,54,55]. The reduction of SnO_2_ to Sn at 0.8–1.2 V vs. Li^+^/Li (green zone 1) is clearly distinguishable for the first cycle and then only very feeble in the next discharge cycles. The alloying process of Sn to Li*_x_*Sn at 0.2–0.4 V vs. Li^+^/Li shows a long and sloping voltage plateau (green zone 2) at the first discharge cycle. On the other hand, the discharge capacity at the second cycle (green line) recovered to 773 mAh g^−1^. The reduction of SnO_2_ to Sn at 0.8–1.2 V is weak but the alloying process of Li_x_Sn_y_ (4.4 Li^+^ insertion) at 0.2–0.4 V vs. Li^+^/Li is dominant. The 20th discharge curve shows the major alloying activity of Li*_x_*Sn. The sloping plateau is characteristic of a solid-solution type reaction, indicating that lithiation occurs homogeneously through the nanoparticles [56,57]. There is a small sloping plateau around 0.1 V vs. Li^+^/Li (red zone), which can probably be ascribed to the lithiation of the amorphous carbon. Indeed, Dahn et al. found also that amorphous carbon contributes to the capacity in the Sn–Co–C alloy [58].

The charge capacity at the first cycle reached 745 mAh g^−1^, recovering to 49% of the first discharge capacity (in red color no. 1). The dealloying process at 0.4–0.6 V (blue zone) is significantly reduced but is yet more pronounced than the alloying process. However, this process remains at the 20th charge curve. Low Coulombic efficiency at the first cycle has been observed for other carbonaceous anode materials such as graphite and graphene due to the SEI formation [59,60,61]. As shown in Figure 7c, the capacity becomes more stable after the 10th cycle with the Coulombic efficiency at >98%. The alloying and dealloying processes are thus retained for the entire 20 cycles.

Figure 7d shows the specific capacities of the SnO_2_@C electrode at different C-rates of C/10, C/7.5, C/5 and C/2. The SnO_2_@C electrodes provided specific capacities of 789 and 649 mAh g^−1^ for C/5 and C/2, respectively. The recovered capacity at C/10 was 761 mAh g^−1^ after a few cycles. Aware of the theoretical capacity of 781 mAh g^−1^ for the alloying/de-alloying reaction, Sn + *x*Li^+^ + *x*e^−^ ↔ Li*_x_*Sn (0 ≤ *x* ≤ 4.4), the SnO_2_@C electrode thus reached almost full capacity at C/10 after several cycles.

The electrochemical characteristics of the SnO_2_@C electrode indicate that the homogeneously distributed SnO_2_ nanoparticles are reduced to metallic Sn, which is imbedded in the porous and amorphous carbon spheres, performing a partially reversible reaction. The void space and the nano-SnO_2_ particles in nano-rattle SnO_2_@C composite accommodate for the volume expansion of Sn and SnO_2_, leading to a stable cycle life.

Another important property of lithium ion batteries is their high volumetric capacity (mAh cm^−3^) for reaching high-energy density, which is often neglected. The volumetric capacity of alloy-based anode materials is even more crucial because of their huge expansion of the lattice volume during lithiation [13,62]. Although the void of the hollow carbon spheres in nano-rattle structures allows to prevent cracks in the electrode, it decreases the electrode density (mg cm^−3^) and areal mass loading (mg cm^−2^), resulting in low areal and volumetric capacities. Therefore, increasing the number of SnO_2_ active particles per carbon hollow sphere is important to raise the areal capacity (mAh cm^−2^ = mAh g^−1^ × (g cm^−2^)). The attempt to synthesize Sn@C by the reduction of Sn^2+^ ions with reducing agents like NaBH_4_ in a carbon matrix leads to only few Sn particles as shown in Figure S4. The electrochemical behavior of nano-Sn@C has been shown in Figure S5. As seen in the CV results shown in (a), the oxidation (dealloying Li-Sn) and reduction (alloying Li -Sn) of the nano-Sn have occurred at 0.5 and 0.2 V vs. Li^+^/Li, respectively. The loss in capacity was very rapid within the first three cycles, and the rate capability of Sn@C was not as good as that of SnO_2_@C (Figure S5c,d). In the end, the Sn@C electrode reached the areal capacity of about 1 mAh cm^−2^. In comparison, SnO_2_@C obtained via melt diffusion gave an enhanced SnO_2_ mass loading and a higher areal capacity (2.1 mAh cm^−2^). Taking into account the density of Sn at 7.3 g cm^−3^ and the one of SnO_2_ at 6.95 g cm^−3^, it is reasonable to make a direct comparison of mass loading.

Increasing the loading of SnO_2_ in SnO_2_@C composite results in high areal capacity, thereby leading to high volumetric energy density. Such a higher loading can be achieved by adjusting the amount of SnCl_2_·2H_2_O precursor along with the increase in dwelling time after thermal treatment.

## 4. Conclusions

Nano-rattle SnO_2_@C composite has been synthesized using reverse microemulsion silica template and molten salt melt diffusion impregnation. This method featured homogeneously distributed SnO_2_ nanoparticles of <10 nm inside and on the porous carbon spheres of 80–100 nm. This nano-rattle SnO_2_@C composite enabled to sustain the effects of volume change due to void space and the formation of nanoparticles of SnO_2_ on porous amorphous carbon. This structured SnO_2_@C exhibited a high reversible process of alloying/dealloying at around 0.5 V vs. Li^+^/Li with a specific capacity of 761 mAh g^−1^ at C/10, which is 97% of theoretical capacity (781 mAh g^−1^) at 4.4 Li storage per Sn atom. On the other hand, this composite showed a partially reversible process of SnO_2_ reduction at 0.8 V vs. Li^+^/Li. Therefore, we showed that the strategy of the formation of SnO_2_@C is promising. We believe that controlling the carbon layer on the silica template, the particles size of carbon and SnO_2_ and the pore size are the critical parameters for high performance.

## Figures and Tables

**Table 1:** BET surface area analysis of (i) SiO_2_@C, (ii) carbon nanocontainers and (iii) SnO_2_@C nano-rattles.

Sample	BET Surface Area (m^2^ g^−1^)	Pore Volume (mL g^−1^)	Mean Pore Size (nm)	Presence of Micro-Pores (<4 nm)
(i) SiO_2_@C	43	0.11	11.4	*x*
(ii) Carbon nanocontainers	167	0.45	8.7	o
(iii) SnO_2_@C nano-rattles	110	0.16	4.7	o

## Supplementary Materials

Supplementary materials: The following are available online at https://www.mdpi.com/2079-4991/10/4/804/s1, Table S1: Calculation of mass of electrode components and volume of NMP solvent to make slurry, Figure S1. TEM and SEM images of silicate using Triton X-100 (a and b) and Igepal CO-520 (c and d) surfactants, Figure S2. Colors of synthetic steps, Figure S3. TEM (a) and SEM (b) images showing SiO2@C after post-calcination thermal treatment, Figure S4. TEM (a), SEM (b) images and (c) EDS analysis of a Sn@C prepared by the reduction of Sn2+ into hollow carbon nanocontainers by a reducing agent of NaBH4, Figure S5. Cyclic voltammetry analysis of Sn@C at 0.05 mV s-1(a). Charge and discharge curves (b) and cyclability (c) of Sn@C composite electrode at C/20 for 3 cycles. Rate capability test of Sn@C electrode at different C-rates for coin cells (d).
