# Supplementary material for: A Nano-Rattle SnO2@carbon Composite Anode Material for High-Energy Li-ion Batteries by Melt Diffusion Impregnation
Source: Nanomaterials (Basel). 2020 Apr 22;10(4):804. doi: 10.3390/nano10040804 (PMC7221675; doi:10.3390/nano10040804)
Supplement: Supplementary file 1 [file nanomaterials-10-00804-s001.pdf]

A nano-rattle SnO_2@carbon composite anode material for high-energy lithium ion batteries by melt diffusion impregnation

Dr. Sivarajakumar Maharajan [1], Dr. Nam Hee Kwon [1,], Prof. Pierre Brodard [2], Prof. Katharina M. Fromm [1,*]*

[1] Department of Chemistry, University of Fribourg, Chemin du Musée 9, CH-1700 Fribourg, Switzerland; sivarajakumar.maharajan@unifr.ch

[2] College of Engineering and Architecture of Fribourg, University of Applied Sciences of Western Switzerland, Boulevard de Pérolles 80, CH-1705 Fribourg, Switzerland; pierre.brodard@hefr.ch

Corresponding Authors

namhee.kwon@unifr.ch (N.H.K.); katharina.fromm@unifr.ch (K.M.F.); Tel.: +41-26-300-87-35 (N.H.K.); +41-26-300-87-32 (K.M.F.) University of Fribourg, Department of Chemistry, Chemin du Musée 9, CH-1700 Fribourg, Switzerland

Calculation of SnO_2 mass in SnO_2@C electrode:

The electrode consists of several components: the active material, binder (Polyvinylidene fluoride) and additive carbon (Super C65). They are mixed in N-methyl-2-pyrrolidone (NMP) as described in the experimental part. The calculation of slurry composition is summarised with an example as shown in table 1. The overall composition is 100% which comprises 77% active material, 15% additive SuperC65 and 8% PVDF binder. The volume of NMP has been optimized to determine the suitable viscosity.

Mass of active material SnO_2@C (mg)	Mass of Super C65 (mg)	Mass of PVDF binder (mg)	Tap density of SnO_2@C (g/mL)	Tap density of Carbon C65 (g/mL)	Tap density of PVDF (g/mL)	Volume of NMP (mL)	Mass of Cu foil disc (mg)	Mass of slurry coated Cu foil disc (mg)	Mass of loading (mg)
100	19.48	10.38	0.75	0.117	0.26	0.34 mL	22.3	18.9	3.4
			Volume: 0.133 mL	Volume: 0.166 mL	Volume: 0.039 mL				

Table S1: Calculation of mass of electrode components and volume of NMP solvent to make slurry

The mass of loading on the electrode discs is calculated by the difference in mass before and after loading. To calculate the mass of SnO_2@C in the composite, excluding carbon C65 and PVDF binder, the following calculation has been done:

Mass of loading (77% SnO_2@C) = ~ 2.6 mg

Further, to calculate the mass of SnO_2 in SnO_2@C nano-rattles, the mass percentage (76%) found in TGA analysis is applied (figure 6(d)). Accordingly,

2.6 * 76% = ~ 2 mg

The mass of SnO_2@C loading in 16 mm disc electrode is found to be ~2 mg. The gravimetric capacity (mAh/g) of the SnO_2@C electrode is determined by considering a mass loading of 2 mg.

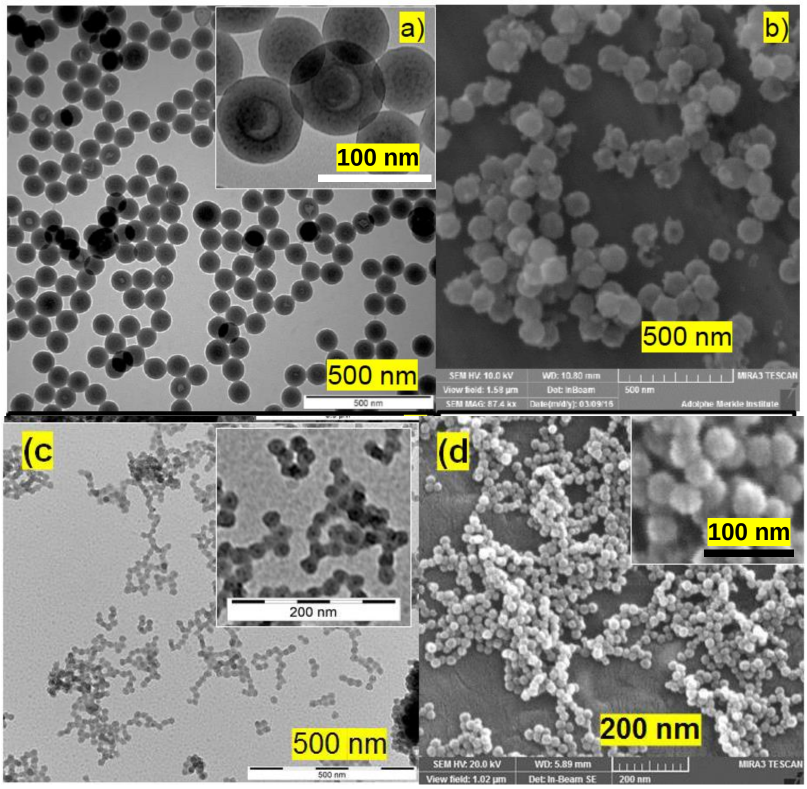

Figure S1. TEM and SEM images of silicate using Triton X-100 (a and b) and Igepal CO-520 (c and d) surfactants.

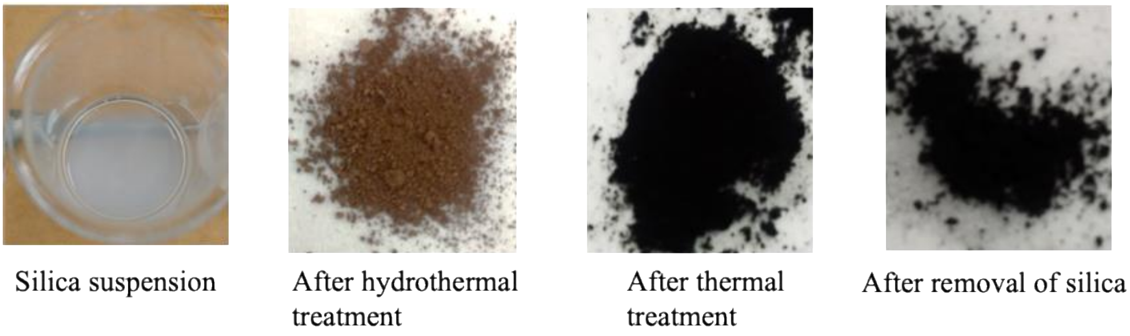

Figure S2. Colors of synthetic steps.

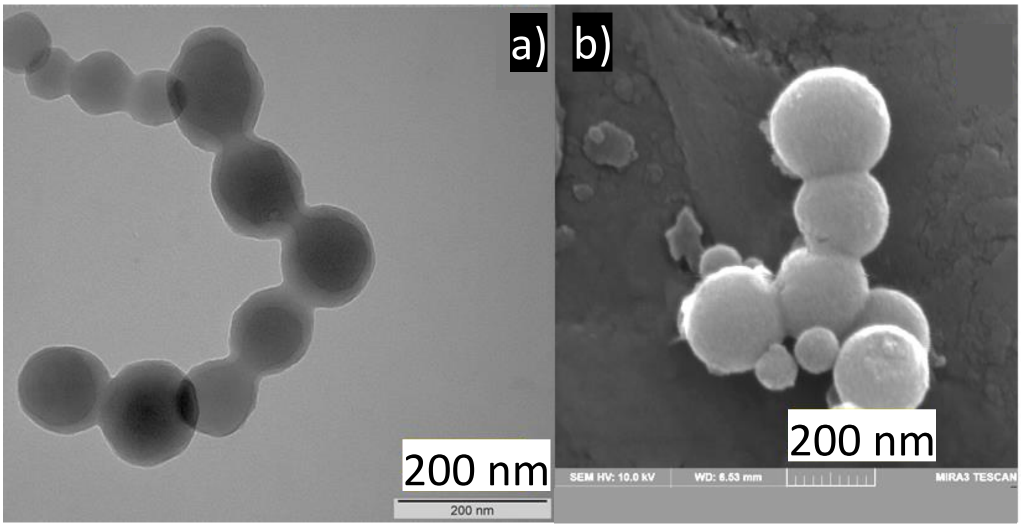

Figure S3. TEM (a) and SEM (b) images showing SiO_2@C after post-calcination thermal treatment.

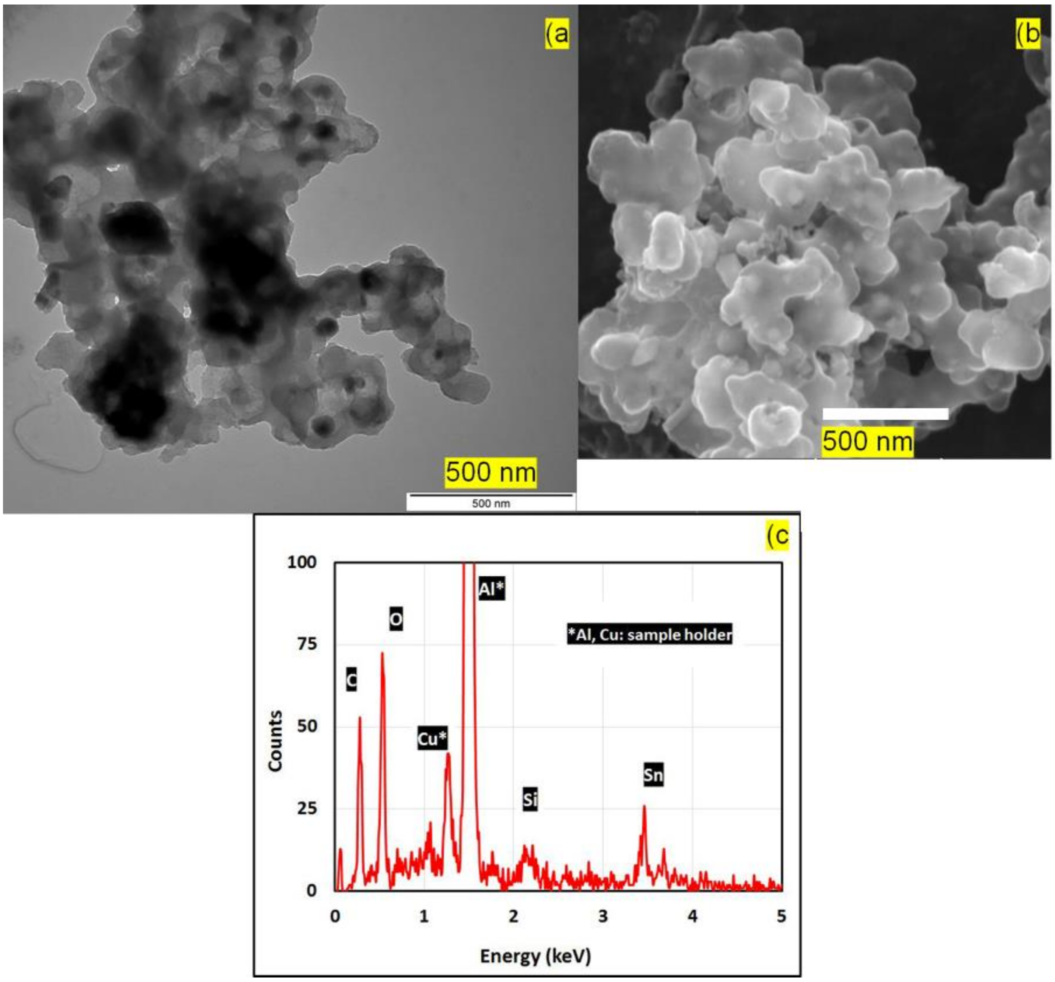

Figure S4. TEM (a), SEM (b) images and (c) EDS analysis of a Sn@C prepared by the reduction of Sn^{2+} into hollow carbon nanocontainers by a reducing agent of $NaBH_4$.

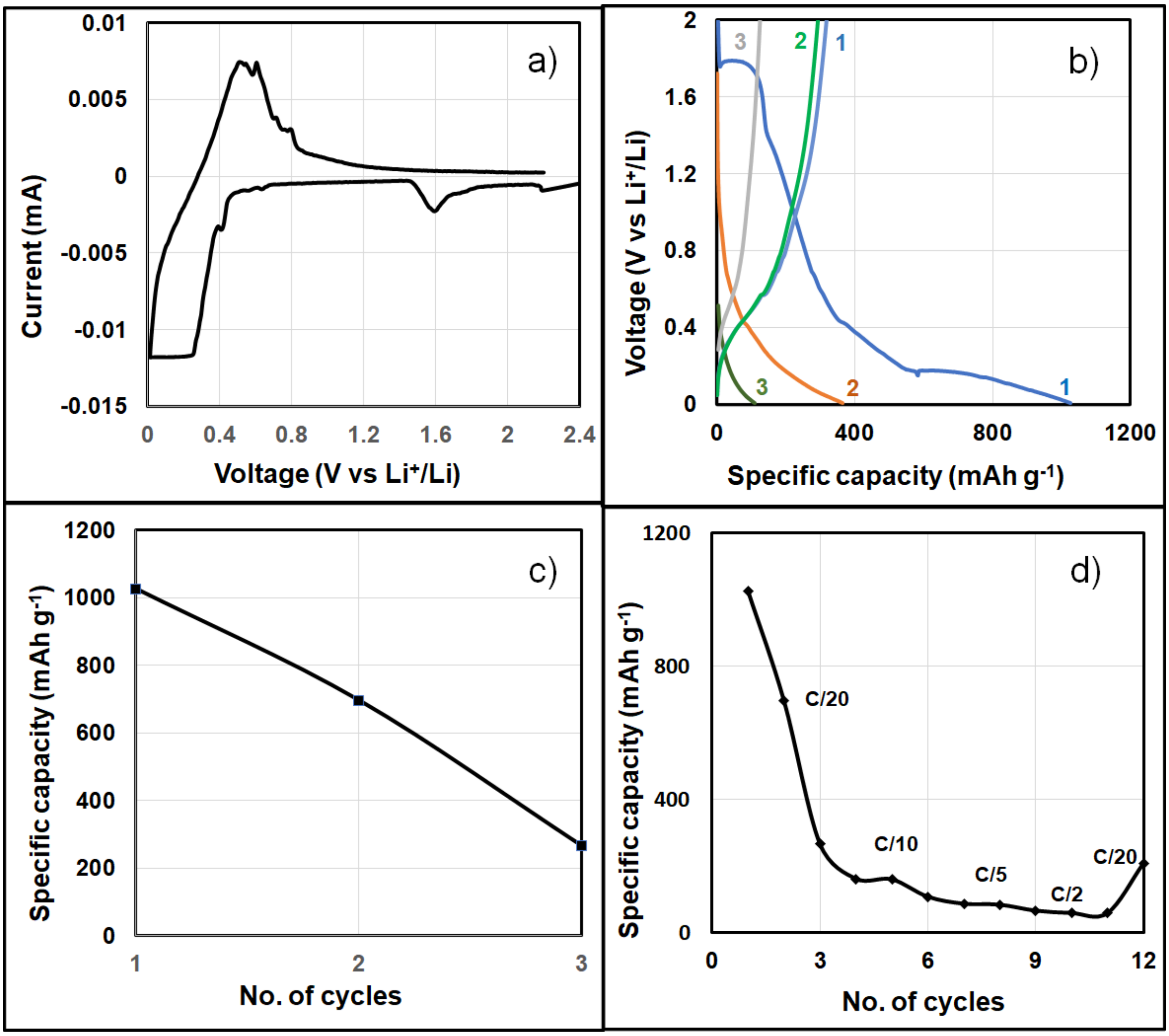

Figure S5. Cyclic voltammetry analysis of Sn@C at 0.05 mV s^{-1}(a). Charge and discharge curves (b) and cyclability (c) of Sn@C composite electrode at C/20 for 3 cycles. Rate capability test of Sn@C electrode at different C-rates for coin cells (d).
